# Deaf children’s experiences of hearing in everyday life: a systematic review

**DOI:** 10.1093/jdsade/enaf035

**Published:** 2025-05-19

**Authors:** Ruth Nightingale, Alys Hollyer, Merle Mahon

**Affiliations:** Language and Cognition, Psychology and Language Sciences, University College London (UCL), London, United Kingdom; Language and Cognition, Psychology and Language Sciences, University College London (UCL), London, United Kingdom; Language and Cognition, Psychology and Language Sciences, University College London (UCL), London, United Kingdom

## Abstract

Despite the recognition that deaf children and young people’s (CYP) experiences should be central to practice and policy that promotes inclusion, relatively few studies have explored deaf CYP’s perspectives concerning their everyday hearing experiences. This systematic review identified and synthesized qualitative research exploring deaf CYP’s experiences of hearing in everyday contexts. Searches identified 31 papers that met the inclusion criteria. Using thematic synthesis to analyze findings, four themes were identified. The experience of hearing was individualised and influenced by the sounds that CYP could hear, the environment, and supportive strategies. Challenging listening conditions and strategies to manage the experience of hearing were identified, which may be considered by parents, practitioners and policymakers. However, due to the multiple factors that influence hearing, deaf CYP should be consulted on what they find challenging and the strategies they find helpful. Further research exploring deaf CYP’s experiences of hearing outside of school is needed.

Over the last 20 years, an increasing body of international research has highlighted how structural and systemic barriers within education and wider social contexts can limit deaf children and young people’s (CYP) opportunities to engage in everyday activities (García-Terceño et al., 2023; Oppong et al., 2023; Tsai & Tsai & Fung, 2005). These barriers, including limited access to language and a lack of deaf awareness, can impact deaf CYPs academic achievement, social interaction and emotional well-being. Although reasonable adjustments, including technologies and interventions, can be appropriate and effective in supporting inclusion, policies that recognize structural barriers and clearly address deaf CYPs needs are required (Levesque & Duncan, 2024). As Levesque and Duncan (2024) note, deaf CYP should be placed at the center of educational and social policies, and “*learning from the wisdom of their experiences will help to guide policies and practices”* (p.126). Therefore, examining the evidence from deaf CYPs experiences is needed to inform strategies that ensure the provision of equitable opportunities that will maximise deaf CYPs inclusion and outcomes.

Although historically there has been a quantitative emphasis in hearing research, such as the measurement of deaf CYP’s hearing in the clinic, it is increasingly recognized that audiological tests may not fully reflect everyday hearing (Rapport & Hughes, 2020). For example, Watson and Gregory (2005) reported that CYP with cochlear implants (CIs) responded to sounds in the clinic, but found increased background noise intolerable in everyday environments leading to rejection of their CIs. Consequently, the need for ecologically valid studies to increase understanding of deaf CYP’s real-life hearing-related function, activity and participation in all contexts, has been emphasised, notably by Keidser et al. (2020). Richer understanding of deaf CYP’s experiences would support both the development of appropriate and individualised interventions and would also facilitate improved methods for assessing and predicting a deaf CYP’s ability to accomplish real-world tasks (Keidser et al., 2020). In their study of deaf adults, Pryce et al. (2024 p.8) concluded that *“audiological care should include a recognition of the individualised experience of hearing loss and explicit provision to building support”*.

More often, experiential studies generally use quantitative data from survey reports made by deaf CYP, parents, and teachers. Using quality of life measures with deaf CYP and their parents, Fellinger et al. (2008) found parents were more likely to have a positive view of their child’s quality of life, compared to their child’s responses. The self-rated scores from CYP demonstrated a more complex picture, with higher rates of dissatisfaction being reported in relation to interests, recreational activities, and physical health. These different ratings suggested parents were potentially unaware of their child’s experiences of isolation and reduced physical well-being (Fellinger et al., 2008). More recently, Zhang et al. (2021) found strong correlation between quality of life responses from deaf CYP and their parents in regard to behaviour and emotional well-being. However, weak correlation was found between deaf CYP’s and parents’ responses around the difficulties experienced in different hearing environments, with parents appearing to underestimate the impact of the environment on their child’s hearing. In studies that compared deaf CYP to their hearing peers, higher rates of emotional and behavioural issues were identified among deaf CYP, in particular affecting peer relationships (Stevenson et al., 2015). Despite the literature suggesting deaf CYP may encounter more difficulties in daily life, there is a gap in the evidence base concerning deaf CYP’s experiences, in particular studies that have explored deaf CYP’s own perspectives of everyday hearing and listening.

Rapport and Hughes (2020) argue that because of their focus on people’s lived experiences, qualitative methodologies are critical to addressing this knowledge gap in hearing research. Qualitative research investigating deaf CYP’s experiences has been conducted, although studies have tended to focus on single subsets of the deaf population, such as the experiences of CYP with CI, or deaf adolescents, or on a single context, such as deaf CYP’s experiences at school. Consequently, deaf CYP’s experiences of everyday hearing is not understood holistically, across a range of ages, hearing device use, educational settings and other everyday contexts. This highlights a gap in knowledge and the need for research that reflects the diversity of deaf CYP’s experiences. Increased understanding of deaf CYP’s hearing in everyday situations, including what makes listening difficult and the factors that interact with hearing in real-life function, including systemic barriers, could potentially identify the support and interventions needed to reduce the academic, social and emotional challenges that deaf CYP may face (Keidser et al., 2020). In addition, an understanding of deaf CYP’s lived experiences of hearing would contribute to development of policies and practice that *“acknowledge belonging and participation, self-determination, self-advocacy, and identity as essential aspects of inclusion”* for deaf CYP (Levesque & Duncan, 2024 p.126).

To address this gap in understanding, inform future qualitative research of deaf CYP’s experiences, and provide evidence to help guide inclusive practice and policy, a review of the existing literature is necessary. A systematic review, using recognized methodology and critical appraisal to avoid bias, was chosen in order to answer a specific research question, rather than a scoping review which would have provided an overview of the topic (Munn et al., 2018). By identifying and synthesizing the findings from qualitative studies exploring deaf CYP’s experiences in real-life situations, this systematic review aimed to answer the question: what are deaf CYP’s experiences of hearing in everyday life?

## Methods

### Study design

A qualitative systematic review was conducted to synthesize the experiences of everyday hearing for deaf CYP. Systematic reviews and syntheses of qualitative studies bring together data from different contexts, identify research gaps, inform future primary studies, and can provide evidence for practice and policy (Tong et al., 2012). Therefore, it was an appropriate method for addressing the aim of this systematic review. Searching on Open Science Framework, Google Scholar and the International Prospective Register of Systematic Reviews (PROSPERO) found no existing or planned systematic review exploring this topic. The Preferred Reporting Items for Systematic reviews and Meta-Analyses (PRISMA) (Page et al., 2021) was used to conduct and report this review. The review protocol was registered on PROSPERO (CRD42024505577) (Hollyer et al., 2024).

### Eligibility criteria

The SPIDER (Sample, Phenomenon of Interest, Design, Evaluation, Research type) tool for qualitative evidence synthesis was used to develop the inclusion and exclusion criteria (Table 1) (Cooke et al., 2012).

**Table 1:** Inclusion and exclusion criteria.

Criteria	Inclusion	Exclusion
Sample: participants	CYP aged 0-18 Studies involving stakeholders (e.g., parents, teachers) that reference CYP’s experience of hearing	Mean age of CYP is reported as over 18 years of age Studies where stakeholders are reporting on their own experiences
Sample: condition	Deaf, including unilateral or bilateral, mild-profound, permanent or temporary hearing loss Deaf CYP with language disorders as deaf CYP often have atypical language skills associated with their hearing (Sarant et al., 2009)	Deaf CYP with additional sensory, physical or learning needs which impact daily life
Phenomenon of Interest	Deaf CYP’s experiences of hearing in everyday life	Studies that do not report on deaf CYP’s experiences of hearing
Design and Research Type	Primary qualitative research published in peer-reviewed journals	Quantitative or mixed methods designs. As the aim of the review was to explore the complexity of CYPs experiences, only standalone qualitative studies were included as it was more likely these studies would provide the “thicker” descriptions, rich data and more in-depth analysis needed to conduct a thematic synthesis Non-peer-reviewed publications, reviews, discussion papers, dissertation/thesis, conference papers, and pre-prints
Date range	Published between 2001-2024. The newborn hearing screening program was introduced in the UK in 2001 which contributed to the earlier detection of moderate and profound hearing loss (Neumann et al., 2022)	Published before 2001
Language	Published in English	Not available in English

### Search strategy

A preliminary search of Medline was undertaken to identify articles on the topic. The text words contained in the titles and abstracts of relevant articles, and the index terms used to describe the articles were used to develop a full search strategy. The PRISMA checklist for literature search reporting (Rethlefsen et al., 2021) and the SPIDER tool (Cooke et al., 2012) were used to develop the search strategy, in consultation with a university librarian. The search strategy was adapted for each included database. Medline, ERIC, PsychINFO, British Educational Index, Web of Science, and CINAHL databases were searched. Where possible, search results were filtered for English language and year of publication. Supplementary material 1 shows the search strategy used in Medline. The final search was completed on 6^th^ February 2024. In addition, all references from included studies were screened.

### Study selection

Search results were exported, de-duplicated and screened using Covidence systematic review software (2024). Following a pilot test, two reviewers (RN and AH) independently screened titles and abstracts using the inclusion and exclusion criteria. Any disagreements were resolved through discussion with the third reviewer (MM). Full text articles were each independently reviewed by two reviewers (AH and either RN or MM) against the inclusion criteria. Disputes were discussed by all three reviewers. Reasons for exclusion of articles at full text that did not meet the inclusion criteria were recorded.

### Data extraction

One reviewer (AH) conducted data extraction using a template in Covidence designed specifically for this review. Following piloting of the draft extraction form, data extracted included research aim, study design, country, participants, main findings, theory and patient and public involvement (PPI). The content of data extraction forms was reviewed and discussed by all authors.

### Quality appraisal

The Critical Appraisal Skills Programme (CASP) checklist for qualitative research (CASP, 2023) was used in Covidence for quality appraisal of included studies. Quality assessment was completed by AH and a second reviewer (RN) independently appraised 20% of included articles. Any disagreements were discussed with MM. No studies were excluded based on the quality assessment, although this was taken into account during synthesis.

### Data synthesis

Thematic synthesis (Thomas & Harden, 2008) was used as it provides a flexible, systematic and transparent method to move from the findings of multiple qualitative studies to synthesis (Booth et al., 2016). One reviewer (AH) coded the findings of each study inductively and line-by-line using Taguette software (Rampin & Rampin, 2021). This involved reading each sentence or paragraph carefully and assigning it with one or more codes that captured the meaning or content of the text. Coding each study involved “translation” (Thomas et al., 2017), where concepts and codes were compared across all the studies and judgements made around whether a line of text fitted into existing codes or required a new code to be generated. Before completing this stage of the synthesis, codes were re-examined to check consistency of interpretation and to confirm their meaning in the data. To enhance reflexivity and rigour, a second reviewer (RN) independently coded two of the included studies. The similarities and differences between the two reviewer’s codes were discussed by the research team, and codes were then grouped to construct descriptive themes. Through discussion between the three reviewers, analytical themes were developed. As PPI in the conduct of research is recognized as increasing the quality and relevance of studies (Brett et al., 2014), preliminary themes were discussed with a PPI group consisting of nine CYP attending a specialist deaf school. The purpose of this PPI consultation was to explore whether review findings reflected their own experiences of everyday hearing and whether the researchers had missed anything in their interpretation of the data (Walker et al., 2021). Discussions with this PPI group are integrated into the results section.

### Reflexivity and positionality

To ensure trustworthiness and credibility, reflexivity and regular discussion among the three authors occurred throughout the review process. One of the reviewers is hard-of-hearing, and is a speech and language therapist. The other two reviewers are hearing and have professional backgrounds in research with deaf children, speech and language therapy and occupational therapy.

## Results

### Search results

**Figure 1:**
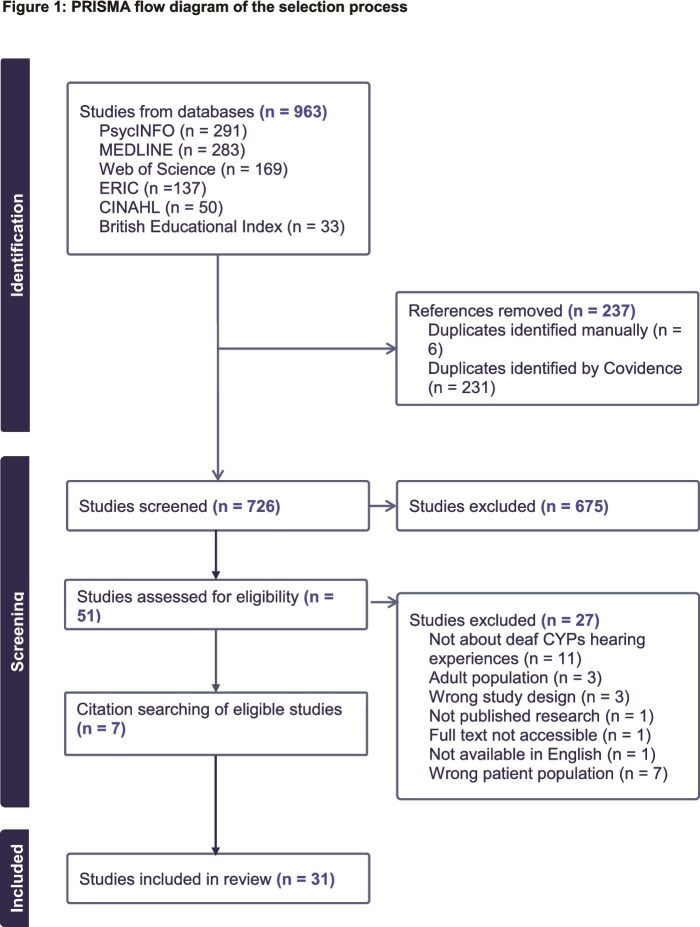
PRISMA flow diagram of the selection process.

Database searching identified 963 articles, of which 237 were duplicates. After title and abstract screening, 675 articles were excluded. Full text screening of 51 articles identified 24 articles that met the inclusion criteria. Hand-searching reference lists of these 24 articles identified a further seven articles. A total of 31 articles were included in this review (Figure 1).

### Study characteristics

Sixteen studies were conducted in the UK, the rest were conducted in Australia, Brazil, Canada, Israel, New Zealand, Sweden and the USA. Fourteen studies regarded CYP with CI’s, two studies focused on CYP with hearing aids (HA), and eight studies involved a mix of CYP using HAs, CIs, Frequency Modulation (FM) systems or no amplification. Seven studies did not specify the type of hearing device used by CYP. Sixteen studies had deaf CYP participants, six had parent participants and nine included a mix of deaf CYP, parents, siblings, teachers and healthcare professionals. Nine studies regarded CYP aged 0-11, 12 studies regarded deaf CYP aged 12-19, seven included a range of ages and three did not specify. Eight studies’ primary aim was to explore experiences at school and an additional six studies included a significant focus on deaf CYP’s academic function. Most studies used semi-structured interviews; focus groups and open-text questionnaires were also used. Studies used a range of analysis methods including thematic analysis and content analysis. Ten studies did not state their analysis method. Four studies used PPI to develop interview schedules. Supplementary material 2 provides a summary of the included papers.

### Quality appraisal

No studies were excluded based on quality. Supplementary material 3 presents a summary of the quality appraisal. All studies used an appropriate qualitative methodology and most had a clear statement of the study’s aims. Common limitations included: not considering the researcher-participant relationship, lack of rigor in data analysis, unclear recruitment strategies and limited reporting of ethical issues.

### Experiences of hearing in everyday life

**Figure 2:**
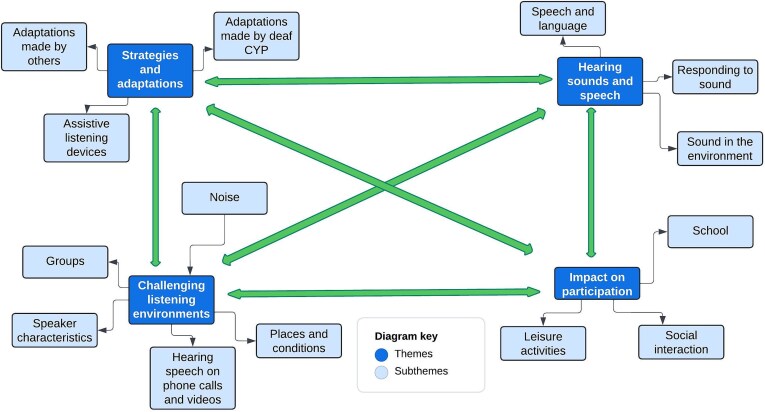
Deaf CYP’s experiences of hearing in everyday life.

Four themes were identified that illustrate deaf CYP’s experiences of hearing in everyday life: (a) hearing sounds and speech (b) challenging listening environments (c) strategies and adaptations, and (d) impact on participation. Bidirectional interactions between the themes highlight how hearing experiences are complex and multifactorial. The themes, subthemes and the interactions between these different aspects of hearing experience are illustrated in Figure 2 and described in detail below.

### Hearing sounds and speech

Experiences of hearing in everyday life were shaped by the sounds and speech that deaf CYP could hear. This included how deaf CYP responded to sounds in the environment, including speech and language. As shown in Figure 2, deaf CYP’s participation in daily activities was influenced by the sounds and speech they could hear.

#### Sound in the environment

Deaf CYP’s hearing of environmental sounds varied considerably. Whereas some deaf CYP experienced difficulties with hearing environmental sounds such as traffic or loud noises (Watson et al., 2016), CYP who had sequential CIs were able to discern sounds they could not hear before their second CI such as aeroplanes, church bells and birdsong (Preisler et al., 2005). Sound localisation varied considerably; while many CYP with sequential CIs reported localisation was easier with two CIs, others reported ongoing difficulties especially in noisy environments (Mather et al., 2011; Mather et al., 2011). Although the use of hearing devices influenced deaf CYP’s hearing of sound, experiences were individualised and influenced by multiple factors including the environment and sound characteristics. By emphasising the individuality of their hearing experiences, the PPI group supported this finding.

#### Responding to sound

Access to sound influenced how deaf CYP responded to their surroundings, which impacted on their participation in everyday activities. Although some CYP could hear traffic noise with their hearing devices, others struggled with this (Mulla et al., 2013; Tierney et al., 2015). Being unable to hear and respond to traffic noise had implications for CYP’s safety and could limit independence in daily life:

*At the minute if he wants to play out and he’ll play out just in front of the house, without hearing aids it wouldn’t happen because he wouldn’t hear the cars coming down. [Parent]* (Tierney et al., 2015, p. 28)

In contrast, parents described how their child responding to their name being called improved their safety (Lin et al., 2022):

*She turns to her name every time, so I can stop her touching something dangerous.* (Sach & Whynes, 2005, p. 403)

Although the PPI group agreed that hearing impacted on awareness of their surroundings, they explained how other senses such as sight and smell were also important. This suggests there are factors additional to hearing that influence deaf CYP’s awareness and response to their environment.

#### Speech and language

Some deaf CYP were unable to hear specific speech sounds, which affected their ability to articulate accurately:

*She sort of has trouble sometime saying her ‘s’ clearly or her ‘sh’ sound or she’ll drop some of the consonants sometimes at the ends of words. I think because she doesn’t hear them herself so it’s hard for her to remember to make the sounds when she’s talking. [Parent]* (Grandpierre et al., 2017, p. 144)

Teachers reported deaf CYP could have difficulty with grammar such as plurals as they were not able to hear the speech sound that marks them (Fitzpatrick & Olds, 2015). Difficulty with spoken language could impact some deaf CYP’s confidence, social interaction and sense of belonging:

*I do find it difficult to make friends because I’m quite a very shy person and I don’t have that much confidence in speaking up to new people. Sometimes when I meet new friends, sometimes they do not understand me because, obviously, when I was born, I was not meant to have my voice.* (Terlektsi et al., 2020, p. 159)

The PPI group supported and challenged this finding that hearing sounds and speech influenced deaf CYP’s spoken language and interactions with others. As PPI group members used spoken and/or sign language, they believed effective interactions were dependent on the individual and their communication partner being able to use and understand the preferred communication modality. This suggests that difficulties with spoken language may have a greater impact on the interactions of deaf CYP who only use spoken language compared to those who use sign language. This highlights how hearing experiences are individualised as a result of deaf CYP’s interaction with other factors, such as the listening environment.

### Challenging listening environments

Various aspects of the physical and social environment, including places, activities and situations, were challenging for deaf CYP. Interactions between the environment, strategies, and participation shaped deaf CYP’s experiences of hearing in everyday life (Figure 2).

#### Noise

Background noise was a barrier to participation in daily activities for deaf CYP, regardless of age and hearing device (Bartlett, 2017; Squires & Kay-Raining Bird, 2023). The PPI group agreed that noise was challenging, especially for hearing speech. Noise particularly impacted participation in school, with deaf students reporting that noise levels in mainstream classes prevented them from hearing (Andersson & Adams Lyngbäck, 2022; Bartlett, 2017) and distracted them from the lesson:

*Mainstream classes are quite different, they can be very noisy, where it is very quiet here [in the deaf unit]. The noise makes me forget and find work confusing.* (Iantaffi et al., 2003, p. 153)

Classroom noise caused distress, pain and headaches for some deaf CYP (Lindburg et al., 2021; Todorov et al., 2022). These experiences demonstrate how noise may hinder deaf CYP’s participation in schoolwork and impact their learning.

Experience of noise was influenced by CYP’s hearing device. Some CYP reported headache, nausea and pain related to noise while using their CIs (Watson & Gregory, 2005). For CYP with sequential CIs, some were able to cope better with noise after their second implant (Mather, Gregory, & Archbold, 2011), whereas for others, noise remained challenging:

*Even though you have two implants it’s still sometimes difficult to hear in noisy situations. It’s not 100% efficient. It’s 80% I’d say. With one implant it was 50 or 60%.* (Mather, Gregory, & Archbold, 2011, p. 165)

Although some CYP found their hearing device improved their hearing in noisy environments (Fitzpatrick et al., 2011), others had difficulty in noise as it was amplified by their hearing device. Amplified background noise impacted what the deaf CYP could hear and process:

*What I did hear was like the pens scratching and irritating noise and my brain focused in on those sounds so I didn’t hear what the teacher said.* (Andersson & Adams Lyngbäck, 2022, p. 254)

These experiences highlight the challenges of using hearing devices in noisy environments which meant some CYP removed their devices to decrease noise and discomfort caused by amplification (Mulla et al., 2013; Preisler et al., 2005).

#### Places and conditions

School cafeterias, playgrounds and halls were identified as challenging listening environments for deaf CYP (Tierney et al., 2015). The acoustics of gymnasiums and halls often created echoes which increased noise (Squires & Kay-Raining Bird, 2023). Outdoor playgrounds were considered challenging due to a combination of noise from children shouting, with environmental conditions such as the wind and needing to hear from a distance (Lindburg et al., 2021; Preisler et al., 2005). The PPI group explained how busy environments made it more difficult to lipread or sign; this extends understanding of how places and conditions influenced hearing experiences.

#### Groups

Studies highlighted how groups were often difficult for deaf CYP to participate in, leading to a preference for one-to-one interactions (Hilton et al., 2013; Punch & Hyde, 2011). The PPI group confirmed group conversations were often difficult, due to overlapping speakers. Teachers observed deaf CYP’s difficulty in groups was due to the additional time needed to locate and process who was talking (Fitzpatrick & Olds, 2015). Additionally, increased noise in groups made it more challenging to hear who was talking:

*Too much noise ...when we are doing group work and there’s like five or six different groups in the classroom. That would make it a bit more difficult for me to hear my own group* (Bartlett, 2017, p. 63).

Difficulty hearing in a group made conversation difficult to follow, limiting deaf CYP’s participation and causing frustration:

*I feel annoyed frustrated a little bit upset because I wish I was hearing so I can understand everyone more involved* (Hilton et al., 2013, p. 523).

#### Speaker characteristics

Individual speaker characteristics influenced deaf CYP’s experiences of hearing. Quiet, fast or indistinct speakers were more challenging for deaf CYP to hear and understand (Kent & Smith, 2006); this impacted deaf students’ participation in school:

*When they [the teachers] don’t talk too fast it’s easier for you so you can process what they’re saying, and you can actually get it and answer back.* (Todorov et al., 2022, p. 15)

People wearing dental braces or those with less familiar accents to deaf CYP were harder to understand (Squires & Kay-Raining Bird, 2023). The PPI group identified further characteristics that they found difficult including speech impediments, male voices and lack of familiarity with the speaker. The range of speaker characteristics that individual deaf CYP reported as challenging illustrates the variation in hearing experiences.

#### Hearing speech on phone calls and videos

Some deaf CYP reported hearing speech over the phone was challenging (Archbold et al., 2002; Lindburg et al., 2021). Although many CI users experienced consistent difficulties with using the phone (Punch & Hyde, 2011), some reported improvement following implantation (Hilton et al., 2013), highlighting the variability of hearing experiences among deaf CYP.

Hearing speech when viewing videos was difficult for some deaf CYP (Bartlett, 2017), which impacted their understanding of what they were watching:

*When we are watching television, she says “what was that mom?” for a lot of things. She doesn’t catch everything. [Parent]* (Vieira et al., 2018, p. 219)

The PPI group confirmed this finding, advising that subtitles, in particular those specifically for deaf people, were useful when watching TV and films to avoid missing plot details. The use of strategies and their impact on hearing experiences will be discussed next.

### Strategies and adaptations

A range of strategies and adaptations, including assistive listening devices, were used by deaf CYP and others to manage their hearing experience. The availability and use of these strategies influenced the sounds and speech that deaf CYP could hear and their participation in daily activities (Figure 2).

#### Adaptations made by deaf CYP

Adaptations that improved deaf CYP’s experience of hearing included positioning themselves near the sound source, using non-auditory cues and reducing background noise (Hardy, 2010). Sign language or written cues were used by deaf CYP when spoken information was misheard (Todorov et al., 2022). Some deaf students copied the actions of peers when instructions were missed (Tanure Alves et al., 2021). While many deaf students preferred sitting near the teacher in class to be closer to the sound source and to lipread (Tanure Alves et al., 2021; Todorov et al., 2022), other preferences were reported, illustrating the need for tailored strategies:

*I don’t want to because in the front row you’re too close and I can’t exactly look up the whole time to look at your mouth – so I preferred it in the second row and then the teacher would be like, “No, no, no, you have to sit in the front row” and I had to spend the whole year in the front row* (Bartlett, 2017, p. 63)

However, deaf CYP’s use of strategies and adaptations was dependent on their availability, such as leaving a noisy classroom to work in a quieter space (Edmondson & Howe, 2019). This highlights how the physical and social environment could facilitate or impede deaf CYP’s hearing and inclusion in everyday activities.

#### Adaptations made by others

The adjustments made by others affected deaf CYP’s hearing experiences. Useful strategies included: teachers or peers repeating misheard speech; teachers managing classroom noise levels, and friends talking one at a time (Todorov et al., 2022). The culture of deaf CYP’s environment contributed to how confident they felt asking for adaptations. For example, good relationships with peers, and teachers creating a supportive environment, contributed to deaf students feeling more able to self-advocate (Terlektsi et al., 2020). In contrast, some students felt too embarrassed to give their FM system to the teacher in front of their classmates (Terlektsi et al., 2020), suggesting an unsupportive classroom culture may inhibit deaf CYP asking for accommodations.

Due to limited deaf awareness, deaf CYP’s needs were often misunderstood by classroom teachers (Fitzpatrick & Olds, 2015), including misperceptions that hearing devices negated being deaf or CYP with good speech intelligibility being misconstrued as hearing (Bartlett, 2017; Wheeler et al., 2007). These beliefs influenced adaptation provision (Wheeler et al., 2007), and placed additional strain on deaf CYP when accommodations were not made:

*Sometimes it’s quite frustrating because ... everyone’s like “she hears fine” and everything but...actually it’s quite frustrating sometimes because I have to put in extra effort* (Watson et al., 2016, p. 299)

Some deaf CYP reported a lack of understanding from classmates contributed to prejudice and feelings of isolation, ultimately leading to withdrawal (Tanure Alves et al., 2021). However, being perceived as hearing created a conflict for some deaf CYP who wanted to “fit in” but also needed support:

‘I’m going to treat you as normal, just like everyone else. I’m going to make no allowances for the fact that you’re deaf’. To me, yeah, *that can be a good thing, because you don’t like being seen as different from everyone else but then there are times when actually, yes, you do need those little allowances. [deaf student quoting their teacher]* (Bartlett, 2017, p. 63)

The ambivalence felt by deaf CYP around others’ perceptions of their deaf/hearing identity was recognied by the PPI group, who appreciated not being treated differently to hearing peers, but then experienced difficulties when accommodations were not made.

#### Assistive listening devices

Although FM systems and other assistive listening devices (ALD) improved deaf students’ access in school (Punch & Hyde, 2011), some deaf CYP reported feeling excluded from classroom activities, as devices were either unavailable or used inconsistently (Squires & Kay-Raining Bird, 2023):

*Sometimes they don’t hand the microphone out to the children so the deaf kids can hear, sometimes they forget… so I don’t know what they’re saying* (Todorov et al., 2022, p. 11)

Other negative aspects of ALD were FM systems picking up distracting noises such as teachers talking privately (Bartlett, 2017). Some deaf CYP found it “a hassle” to pass their ALD to multiple people (Kent & Smith, 2006, p. p. 468), or avoided using them as it made them feel different to their peers (Terlektsi et al., 2020). This suggests that the individual deaf CYP’s identity and the culture of the environment shaped how ALDs were used, influencing hearing experiences, feelings of belonging, and participation in everyday activities.

### Impact on participation

Hearing experiences had an impact on deaf CYP’s participation in school, social interaction and leisure activities. As Figure 2 illustrates there were bidirectional effects between the environment, the sounds and speech deaf CYP could hear, and their participation in everyday activities.

#### School

Teachers reported that starting primary school presented challenges for deaf CYP as it involved exposure to new sounds from multiple sources and hearing over significant background noise for the first time (Fitzpatrick & Olds, 2015). Both HA and CI users demonstrated reliance on their devices for school; some refused to attend school without their device, suggesting hearing demands were higher in school than at home (Wheeler et al., 2007). As CYP got older, hearing demands at school increased due to multiple factors including larger and busier school environments, expanding vocabulary and less frequent use of visual cues (Iantaffi et al., 2003). Teachers reported some deaf CYP struggled to understand complex oral instructions, and needed additional support such as repetition, sign or written information to participate in lessons (Doherty, 2012). The additional effort required in class and need for listening breaks was experienced by many deaf CYP (Bartlett, 2017), and parents reported their deaf children were more tired after school compared to hearing children (Mulla et al., 2013). In addition to listening effort and fatigue, the physical impact of increased noise reduced deaf students’ capacity to engage in lessons:

*The classroom gets really noisy and so it gets me like more of a headache and it just makes it harder for me to concentrate so I get more stressed… it stops me from joining in.* (Todorov et al., 2022, p. 10).

The PPI group supported this finding; they described listening and lipreading as effortful and accumulative fatigue meant it was more difficult to concentrate by the end of the week.

#### Social interaction

The hearing demands of interaction appeared to impact deaf CYP’s participation in social activities. For example, some deaf CYP’s participation in play was reduced as they missed invitations or misheard games (Cagulada & Koller, 2020). Parents reported how this could result in their deaf child withdrawing from social interaction or feeling excluded (Tierney et al., 2015):

*He says “Mom it’s hard to socialize. I don’t know what people are saying. I can’t follow the rules of the games. They change the rules on me, and I don’t know that they’ve changed them.”* (Cagulada & Koller, 2020, p. 147)

The hearing demands required for social interactions seemed to change with deaf CYP’s development; for example, conversation-based interactions replacing physical play meant some deaf CYP found it harder to participate in activities with peers as they got older (Punch & Hyde, 2011). As a result, some deaf CYP preferred physical games as they required less spoken communication (Cagulada & Koller, 2020). Different social and physical environments presented challenges for deaf CYP as they got older, including parties and the cinema (Lindburg et al., 2021; Rich et al., 2013). However, the PPI group viewed social interactions to be influenced by additional factors including level of hearing, the environment and acquired skills, suggesting the relationship between hearing demands and age is complex and multifactorial.

#### Leisure

Hearing impacted on deaf CYP’s participation in leisure activities, such as music or sport. While some reported difficulty listening to music (Lindburg et al., 2021), others enjoyed music with friends (Terlektsi et al., 2020). Some deaf CYP showed a preference for physical hobbies, potentially due to reduced demands on their hearing to communicate (Cagulada & Koller, 2020). However, the type of sport appeared to be influenced by deaf CYP’s hearing experiences. For example, the need to remove hearing devices for swimming and increased noise during team sports impacted on deaf CYP’s ability to hear instructions (Grandpierre et al., 2017). Consequently, some deaf CYP preferred solo sports such as cycling (Cagulada & Koller, 2020).

## Discussion

This review has systematically identified, critically appraised, and synthesized qualitative evidence on deaf CYP’s experiences of hearing in everyday life. Four interacting themes were identified that offer new understanding of the complexity and multifactorial nature of deaf CYP’s experiences. The sounds and speech that deaf CYP’s could hear was found to vary, and could be influenced by their level of hearing, hearing device and environment. Although deaf CYP and others used a range of strategies to manage their hearing experiences, use of adaptations was influenced by their availability, deaf CYP’s identity and self-advocacy, misperceptions by others, and the culture of the environment. Deaf CYP’s experiences of hearing influenced their participation in school, social interactions and leisure activities. This suggests that experiences of hearing have a significant impact on many aspects of deaf CYP’s lives, including their real-life functioning, activity and inclusion (Keidser et al., 2020).

This review highlighted how deaf CYP’s participation was influenced by the interaction between the sounds and speech they could hear, and the social and physical environment, which included systemic barriers. Deaf CYP who felt supported in their environment were more likely to feel included, accepted and able to self-advocate. This is consistent with previous research which found that deaf awareness in peers and teachers was positively correlated with social inclusion (Hadjikakou et al., 2008). Misperceptions of deaf CYP as “hearing” and deaf CYP feeling they should “appear normal” despite needing support, appeared to be a barrier to deaf CYP receiving and requesting supportive accommodations (Punch & Hyde, 2005). This highlights the need to develop policies and practices that address the systemic and structural barriers that exclude deaf CYP, such as increasing deaf awareness (RNID, 2024). Deaf CYP may also benefit from additional support to explore their identity, and develop self-determination and self-advocacy skills to use and request accommodations.

Deaf CYP’s hearing experiences at school, such as classroom noise, inaccessible group discussions and fatigue related to listening effort, impacted their participation and learning (Anmyr et al., 2011). These barriers to inclusion at school may provide some explanation for the attainment gap between deaf and hearing students (Hutchinson, 2023) and higher rates of emotional and behavioural issues in deaf CYP (Stevenson et al., 2015). Strategies that improved deaf CYP’s experience of hearing and supported participation were identified, including reducing noise, positioning near the sound source and consistent use of ALDs. However, a survey by the NDCS (2021) found that 68% of UK teachers did not feel confident teaching deaf students, reinforcing the importance of positioning deaf CYP’s experiences at the centre of educational policies to maximise inclusion (Levesque & Duncan, 2024). Findings from this review may inform parents and practitioners of the challenges deaf students face in class and indicate supportive strategies. However, as hearing experiences are individualised and deaf CYP expressed a range of preferences, each deaf student should be consulted as to what accommodations they find helpful.

Deaf CYP’s experiences of hearing impacted their sense of belonging and participation in social and leisure activities. These findings support the existing literature that have identified deaf CYP can find peer relationships challenging (Bat-Chava & Deignan, 2001; Punch & Hyde, 2005) due to deaf CYP’s individual characteristics, peer characteristics and the context of the social interaction (Batten et al., 2014). However, this review extends understanding of the factors that interact with hearing and impact on real-life function (Keidser et al., 2020), such as changes to hearing demands as deaf CYP mature. This suggests the support that deaf CYP receive should adjust to reflect their age and developmental stage. However, as the PPI group suggested other factors interacted with hearing demands as they got older, qualitative longitudinal research is needed to explore how deaf CYP’s hearing experiences change over time and what influences these changes.

### Strengths and limitations

A main strength of this review was its systematic nature; each stage was systematically recorded, discussed and reflected on among the three reviewers. By synthesizing the experiences of deaf CYP of different ages, hearing levels, educational settings and hearing devices, this review has extended understanding of the diversity of deaf CYP’s experiences. However, as deaf CYP’s characteristics were inconsistently reported across the studies (Supplementary material 2), further research is required to explore how experiences differ across these different groups. Although the PPI was conducted with CYP attending a specialist deaf school who would have different experiences to deaf CYP in mainstream settings, this was a valuable exercise that strengthened the quality and relevance of its findings.

This review had some limitations including the requirement for articles to be in English for inclusion, which may have resulted in some relevant non-English studies being omitted. The quality of the included studies was variable (Supplementary material 3). For example, 25 studies did not consider the influence of the researcher affecting confirmability; the recruitment strategy was unclear in 11 studies, impacting the transferability of findings; and only four studies included PPI. Although some of these limitations may relate to the age of the research and differences in reporting standards, it indicates a need for further qualitative research to explore deaf CYP’s experiences of hearing that is methodologically robust and includes PPI. Additionally, many experiences reported in this review were in relation to accessing school. Further research exploring deaf CYP’s experiences of hearing outside of school is required to broaden understanding and consider how hearing impacts deaf CYP holistically.

## Conclusion

This review has highlighted how deaf CYP’s experiences of hearing in everyday life were individual and multifactorial. Challenging listening conditions and strategies to manage the experience of hearing for deaf CYP were identified. These new insights may be considered by parents, practitioners working with deaf CYP and policymakers. However, due to the many factors that influenced their hearing, deaf CYP should be consulted on what they find challenging and the individualised strategies that would help them. The evidence provided by this review could inform the development of policy and practice that removes structural and systemic barriers, and ensure deaf CYP have access to equitable opportunities. Further qualitative research exploring deaf CYP’s experiences of hearing outside of school, including longitudinal studies to explore how experiences change over time, would increase understanding and identify tailored strategies to improve deaf CYP’s hearing experiences, participation and inclusion.

## Supplementary Material

Supp_material_1_Search_strategy_enaf035

Supp_material_2_Summary_of_studies_enaf035

Supp_material_3_Quality_appraisal_enaf035
