# Supplementary material for: Deaf children’s experiences of hearing in everyday life: a systematic review
Source: J Deaf Stud Deaf Educ. 2025 May 19;30(4):446–56. doi: 10.1093/jdsade/enaf035 (PMC12449072; doi:10.1093/jdsade/enaf035)
Supplement: Supp_material_1_Search_strategy_enaf035 [file supp_material_1_search_strategy_enaf035.docx]

**Supplementary material 1: Search strategy used in Medline**

| **Search** | **SPIDER** | **Search Term(s) used** |
| --- | --- | --- |
| 1 | Sample: condition | hearing loss.mp OR exp.hearing Loss/ OR cochlear implants/ OR hearing aids/ |
| 2 | Sample: participants | child* OR young OR teen* OR adolescen* OR p?ediatric |
| 3 | Phenomenon of Interest | listen* OR hearing |
| 4 | Phenomenon of Interest | experience* OR perspective* OR view* OR perception* |
| 5 | Design and Research type | Qualitative OR focus group OR interview |
| 6 | N/A | 1 AND 2 AND 3 AND 4 AND 5 |

Filters: 2001-current, English language
