# Supplementary material for: Deaf children’s experiences of hearing in everyday life: a systematic review
Source: J Deaf Stud Deaf Educ. 2025 May 19;30(4):446–56. doi: 10.1093/jdsade/enaf035 (PMC12449072; doi:10.1093/jdsade/enaf035)
Supplement: Supp_material_2_Summary_of_studies_enaf035 [file supp_material_2_summary_of_studies_enaf035.docx]

**Supplementary material 2: Summary of studies included in the review**

| **Study ID** | **Research aim** | **Study design/ method** | **Country/participant** | **Main findings** |
| --- | --- | --- | --- | --- |
| Andersson 2022 | To explore deaf student's experience of transitioning from mainstream school to a deaf school | Interviews. Thematic analysis. | Sweden. 9 CYP aged 13-17. All used hearing devices but differing amounts. | Many felt a lack of support in mainstream school and although some efforts were made, socially it was challenging. Assistive devices could be challenging due to misuse and distracting due to noises being picked up. CYP use a diverse range of signed and spoken language based on what works for them and their conversation partners. |
| Archbold 2002 | To explore the perceptions of parents of CYP with CIs regarding their child, family, concerns and future needs | Open format text responses to written prompts. Content analysis. | UK. 30 parents of CYP with CIs. | Majority saw increased confidence communicating with hearing and deaf peers, acknowledged that additional support is required. Less need for family as interpreters in conversation. Some CYP are able to monitor their environment using CI e.g. hearing traffic and playing outside. CI needed at school for listening and understanding communication. CYP are able to access activities such as party games and listening to music/watching TV.  Conversation sometimes still challenging environments on the phone. |
| Bartlett 2017 | To explore the classroom experiences of deaf CYP in mainstream schools and the opportunities, barriers and support they receive | Interviews. Thematic analysis. | UK. 10 deaf CYP, age not reported, all secondary school age. Level of hearing or device use not reported. | Radio aids improve access to the lesson, although can be distracting. Increased noise in subjects such as drama makes it harder to hear. Group work is challenging such as being able to hear their own group. Position in the classroom influences hearing. Students have reservations about informing classroom staff they can’t hear. |
| Cagulada 2020 | To explore parents’ perspectives of the social and emotional experiences of their deaf children | Interviews. Thematic analysis. | Canada. 10 parents with deaf CYP aged 3-10. 4 CI users, 5 HA users. | Felt there is a lack of understanding about deaf CYP. Close proximity is important for the child to be able to hear. Social consequences of being deaf e.g. missing out on play, often associated with difficulty communicating. Child express preferences for single sports, potentially due to reduced communicative demands needed to be able to participate. |
| Doherty 2012 | To compare the experiences of deaf CYP and teachers in sign bilingual settings with those who use oral language and total communication | Interviews. Thematic analysis. | UK/Sweden. 36 participants: 8 deaf CYP from Northern Ireland (NI) and Sweden respectively, age 15-23, average age 17.5; 10 teachers at schools for the deaf from each country. | Teachers reported deaf CYP have difficulty with oral instructions; more repetition or use of sign alongside is needed. NI students reported difficulty understanding oral language which hinders their communication and learning from a young age as sign is not taught until later. Swedish students felt able to follow teachers and were supported if they didn’t understand; they related this to the teachers level of deaf awareness and attention given by teaching staff. |
| Edmondson 2019 | To explore the experience of social inclusion of deaf CYP in mainstream secondary schools, including facilitators and barriers | Interviews. Interpretative Phenomenological Analysis (IPA). | UK. 5 CYP aged 13-14 with moderate hearing loss. No information provided about hearing device. | Difficulties in loud environments. Less familiar peers do not understand their experience of deafness, and can express frustration if they have not heard what has been said; they can be perceived as ignorant by others. Sometimes avoided talking to people as it is easier or less awkward. |
| Fitzpatrick 2011 | To explore parents’ perspectives about decision making for, expectations of and outcomes of their child’s bilateral CIs | Interviews. Analysis method not stated. | Canada. 14 parents of CYP with bilateral CI's aged 1-11. | Difficulties with sound localisation, however felt this could be related to age and experience as well as deafness. Understanding of speech and speech in noise improved since second CI e.g. responding to their name in noisy environments. |
| Fitzpatrick 2015 | To understand practitioners’ perspectives about deaf CYP’s communicative, academic and social functioning | Focus groups. Constant comparative method. | Canada. 28 practitioners (17 itinerant teachers, 3 itinerant teachers/ auditory-verbal therapists, 3 audiologists, 4 therapists, 1 speech and language pathologist). | Hearing typically leads to improved communication skills for deaf CYP. Peripheral hearing impacts incidental learning of vocabulary, morphology and general knowledge, therefore deaf CYP have difficulty with these things, applies also to social language. Mishearing can lead to misunderstanding classroom instructions which CYP may not be aware of. CYP that cope well at home can have difficulties in school settings due to increased listening demands. Teachers do not always recognise deaf CYP's difficulties accessing learning such as the impact of a large space on hearing. |
| Grandpierre 2018 | To explore parents’ perspectives on their deaf child’s access to services and how hearing loss has impacted their child’s development | Interviews. Analysis methods not stated. | Canada. 12 parents of 12 deaf CYP aged 7-10 (9 HA users, 2 FM system users, 1 using no device). | Children found noisy environments difficult and overwhelming. Teachers can underestimate deaf CYP’s needs such as positioning in the classroom or need for amplification. Children can find sports noisy and overwhelming or limit hearing as the child needs to take out HAs for safety. Child’s hearing affects their speech as they cannot hear fricative sounds to self-monitor. |
| Hardy 2010 | To explore severe-profoundly deaf adolescents’ deaf identity | Interviews. Grounded theory. | UK. 11 CYP  aged 13-16. No information about level of hearing or hearing device. | Difficulties with hearing when not face to face with the speaker, particularly in noisy environments. Use strategies such as positioning themselves to be able to lipread the speaker. Easier to understand teachers who speak clearly and act when teaching. |
| Hilton 2013 | To explore deaf CYP’s perspectives on decision making for SCI, their identity, and the impact of SCI on their wellbeing | Interviews. IPA. | UK. 11 CYP  aged 12-18 with bilateral CIs. No information provided about education settings. | Being able to localise sound and hear in noise leads to improved communication in group conversation and sports. Able to engage in conversation with reduced instances of mishearing, however there is frustration when others do not repeat themselves. Able to use the phone. Felt excluded when they cannot hear or understand, particularly in group conversations. |
| Iantaffi 2003 | To explore deaf CYP’s experience of inclusion and identify barriers and facilitators to deaf CYP's inclusion in mainstream schools | Interviews, focus groups. Analysis method not stated | UK. 83 students (61 deaf students, 22 hearing) aged 11-14 at mainstream schools. 21 profound HL, 17 severe HL, 19 moderate HL and 4 CIs users. Of the deaf participants, 27 used sign language, 34 used oral language. | Helpful actions of teachers e.g. taking microphones off when not directing the whole class, moving so students can see their face when teaching. Unhelpful actions of teachers make lessons less accessible e.g. talking quickly, not using amplification devices. Challenging listening environments e.g. large spaces with an echo, technology rooms that interfere with amplification equipment. Difficulties making friends due to not being able to hear speech |
| Kent 2006 | To explore adolescent's experiences of using HAs | Interviews. Analysis method not stated. | New Zealand. 16  CYP with HAs aged 12-17. | Microphones are used to support access to lessons; CYP frustrated at reliance on them and needing to pass them to each teacher. Strategies CYP use when they cannot hear include: asking for repetitions, lip reading, amplification, trying to guess what was said. Helpful strategies others use include: getting the persons attention, facing them, assisting with technology. Challenging hearing environments include noise, quiet speakers, lack of repetition from others. |
| Lin 2022 | To explore the experiences of parents managing their child’s hearing loss | Interviews. Content analysis. | Australia. 12 parents of deaf CYP aged 13-29 months (11 HAs). | HAs improved access to sound and therefore better attention and response to sound. More aware of when their child can hear them. Negatives of HAs - feedback from device. |
| Lindburg 2021 | To explore experiences and situations that affect quality of life in deaf CYP | Interviews and focus groups. Thematic and content analysis. | USA. 28 participants:  6 CYP aged 5-7. (3 using HAs, 1 using CIs, 1 using no amplification, 1 hearing sibling); 12 parents of deaf CYP; 10 professionals (5 audiologists, 3 teachers, 2 family coordinators). | Expressed frustration when they are not able to hear what peers can. Struggle in noisy environments and background noise, reported pain and anxiety. Deaf CYP felt they could do most things peers could do; however, significant others reported interactions were limited to a few children.  Limitations in activities included movies, music, using the phone, and group activities. Experienced fatigue, thought to be due to energy used to hear. |
| Mather 2011a | To explore the perspectives of parents and teachers of CYP who receive SCI and their experiences on the outcomes of SCI | Interviews. Analysis method not stated. | UK. 24 participants (12 parents, 12 teachers of CYP aged 10-18 with SCI) | Improvements in CYP’s localisation and hearing from a distance, meant required less repetition from others, use lip-reading less and engaged in group conversation more. Continue to have difficulty with background noise, but less than before SCI. Some CYP appeared less tired, possibly due to less effort needed to hear. |
| Mather 2011b | To explore CYP experiences of sequential CIs (SCI) | Interviews. Analysis method not stated. | UK. 15 CYP aged 10-18 who had a SCI. | CYP found localising sounds and group conversations easier to follow as they can isolate who is talking. SCI facilitated access to environmental noise e.g. car engines, footsteps. Increased background noise hard to deal with or distracting. Participation in leisure activities e.g. listening to music, team sports have improved. |
| Mulla 2013 | To explore parent and CYP perspectives on bone anchored hearing aids (BAHA) | Individual and family group interviews. Thematic content analysis. | UK. 14 participants:  11 parents of CYP using BAHA and 3 CYP. CYP aged 2-23 (average age 9.4 years). | Parents reported that their children are more fatigued compared to other children and felt that their child’s needs are often misunderstood/not acknowledged by others. Parents reported that BAHA improved their child’s awareness of traffic and clarity of sound. Noisy environments such as parties, restaurants or high levels of wind are difficult. |
| Preisler 2005 | To explore the experiences of CYP with CI of the procedure, hearing after implantation, speech and language and peer relationships | Interviews. Analysis method not stated. | Sweden. 11 CYP with CIs aged between 8-10. 5 attended mainstream schools, 6 attended schools for the deaf - interviews conducted in sign language. | Wanted to be surrounded by sound to be aware of their surroundings but occasionally longed for silence. Able to hear environmental sounds such as birds, church bells and traffic following implantation. Sound can be distressing or irritating, at which time they turn the processor off. Difficulty understanding teachers who talk too quickly, too much or too indistinctly.  Difficulty understanding other CYP in the class due to low volume or background noise or in the playground due to screaming. |
| Punch 2011 | To explore CYP with CIs social and emotional development from their own, parents and teachers’ perspectives | Interviews. Constant comparative method. | Australia. 51 participants: 25 parents with child with CIs aged 1-25 (mean age 10). 15 teachers. 11 CYP with CI aged 10-17. | Group conversations are more difficult, one to one is easier. Noise is challenging e.g. playgrounds, big groups. Parents and teachers reported social situations become more challenging with age as talking increases and play decreases and the environments become noisier. Social impacts of being deaf e.g. mishearing conversations, jokes and social situations involving more talking with age. Difficulty using the phone such as difficulties recognising voices and what is being said. CYP felt conscious of their speech and using the FM system in class. |
| Rich 2013 | To explore adolescent CI users’ experiences of educational and social functioning and their hearing/ deaf identity | Open-ended questionnaire. Thematic analysis. | Israel. 12 CYP with CIs aged 14-18. 42% bilaterally implanted  All Hebrew speaking with expressive language skills. | Most CYP able to understand what is said by teachers and peers in class. Those with sequential CI's said this improved their understanding of speech in class. Some CYP reported difficulty with group discussions, others felt able to join group discussion but this got harder with background noise. Quiet surroundings are easier and increasing background noise is harder. |
| Sach 2005 | To explore the expectations and perspectives of parents of children with CI | Interviews. Analysis method not stated. | UK. 216 parents of CYP with CIs. Age of CYP not reported but all attending paediatric CI centre. | Child is more able to engage in family discussions following implantation and less interpreting is needed; many reported increased confidence. Child could hear sounds such as the sea, bird song, and doorbells following implantation. CI had improved safety e.g. hearing their name to stop at a road. |
| Squires 2023 | To explore deaf and hearing CYP experiences of hearing and understanding at school | Interviews. Thematic analysis. | Canada. 32 participants: 16 deaf CYP aged 7-12. 16 hearing CYP aged 7-11. | Deaf CYP reported background noise, amplified by hearing aids, makes speech harder to hear and harder to hear when noise levels in the classroom are not managed. Environments deaf CYP found challenging to hear in include the gym, music room, cafeteria, hallway, playground or bigger rooms due to distance and echoes. Characteristics that make it harder to hear a speaker - low volume speech, retainers/braces or additional languages/accents. FM systems are not always available or inconsistently used in class. |
| Tanure Alves 2021 | To explore deaf CYP's experiences of inclusion in physical education (PE) classes | Interviews. Content analysis. | Brazil. 7 deaf YP aged 17-20 (average age 18), at mainstream school. | Often copied their classmates as they did not understand the teacher’s instructions. Would be able to participate more in PE if their teacher stood closer to them to give instructions or gave them instructions separately. Students felt prejudiced against which contributed to difficulty communicating and participating in sports. |
| Terlektsi 2020 | To explore deaf CYP's experiences of peer relationships | Interviews. Thematic analysis. | UK. 30 deaf CYP aged 13-19 (mean age 16). 21 CYP using HAs, 9 using CIs | Had good relationships with peers and therefore could ask for help when required, good relationships related to feeling accepted. Some had low confidence talking to new people due to difficulty being understood. Some disliked FM systems, potentially due to feeling different. One participant used music to connect with friends. |
| Tierney 2015 | To explore the perspectives of children and parents of children with hearing loss about their daily life | Interviews. Thematic analysis. | UK. 65 participants:  31 parents of 37 CYP with otitis media with effusion (OME) related to cleft lip/ palate. 22 children aged 6-11 with OME related to cleft palate /lip. | Children with HAs reported frustration and distress as they give feedback, amplify background noise or run out of battery, some turn HAs off to manage noise. Environments such as playground, dining hall and hall challenging because of background noise, this impacted play as hard to hear. Play is affected, not able to play outside without HAs or go too far due to danger of not hearing traffic or their own name. Challenges going to the cinema, either can’t hear dialogue or too loud if HA is worn. |
| Todorov 2022 | To explore the barriers and facilitators to engagement for deaf students in mainstream schools | Interviews. Thematic analysis. | Australia. 16  CYP aged 9-12 with bilateral moderate-profound hearing loss (5 with bilateral CI's, 3 HA and CI, 8 HAs) | CYP had difficulty in noise, particularly environments such as gyms and halls. Physical effects of noise include headaches, which CYP associated with difficulty concentrating and increased stress. CYP used strategies such as sitting near the front of class, captions, sign, written information. Strategies that teachers use include: talking clearly and loudly, ensuring the class is quiet for instructions, repeating, positioning themselves so the CYP can hear. CYP used assistive amplification systems but important its correctly use by all in the classroom. |
| Vieira 2018 | To understand the experiences of the family of and child with CI, difficulties, coping strategies and facilitators | Interviews. Grounded theory. | Brazil. 32 participants representing 9 families:  9 CYP with CI aged 6 -11; 9 mothers, 7 fathers, 3 siblings, 5 extended family members. | Parents reported that deaf CYP’s difficulties are often misunderstood by others such as teachers. Difficulties hearing over the phone, understanding speech on TV, hearing far away sounds and understanding new words. Position and noise in the classroom impacts how well they can hear their teacher. Families made adaptations for deaf child to be able to engage in activities. |
| Watson 2005 | To explore CI non-use with children with CI and their parents | Interviews. Analysis method not stated. | UK. 8 participants:  4 hearing parents, 4 CYP with CIs, (ages of CYP not reported). | CYP reported that noise from CI was too loud causing discomfort and pain.  Parents reported that children became frightened of noise. CYP showing response to sound and tolerating CI in clinic but could not tolerate in everyday environments. |
| Watson 2016 | To explore the  experiences of teenagers with CIs and the impact it has on their life | Interviews. Thematic analysis. | UK. 9 CYP with CI, aged between 14-16. | Greater wellbeing following implantation due to ease of interaction with their surroundings and with others. Greater access to environmental sounds such as birds, music and train announcements. Although they functioned and were perceived as hearing, this raised a conflict of liking not being treated differently but still needing accommodations. |
| Wheeler 2007 | To explore CYP with CI's perspectives about the impact of their CI, social and educational abilities and challenges, and identity | Interviews. Analysis method not stated. | UK. 29 CYP with CIs aged 13-16 years. Various school settings including: mainstream, resourced mainstream, school for the deaf. | Feel upset or anxious if they are not able to use CIs. Improvements after implantation e.g. interaction with others, speech production, group discussion and using the phone. Noise, crowds or very quiet environments are challenging. Misperceptions about deafness and being perceived as hearing presented conflicts for CYP; some identified as hearing but needed accommodations. CI improved their hearing but additional support, such as a teaching assistant is required to understand in lessons. |
