# Supplementary material for: Deaf children’s experiences of hearing in everyday life: a systematic review
Source: J Deaf Stud Deaf Educ. 2025 May 19;30(4):446–56. doi: 10.1093/jdsade/enaf035 (PMC12449072; doi:10.1093/jdsade/enaf035)
Supplement: Supp_material_3_Quality_appraisal_enaf035 [file supp_material_3_quality_appraisal_enaf035.docx]

**Supplementary material 3: Quality appraisal summary (n = 31)**

| **Study ID** | **1. Was there a clear statement of the aims of the research?** | **2. Is a qualitative methodology appropriate?** | **3. Was the research design appropriate to address the aims of the research?** | **4. Was the recruitment strategy appropriate to the aims of the research?** | **5. Was the data collected in a way that addressed the research issue?** | **6. Has the relationship between researcher and participants been adequately considered?** | **7. Have ethical issues been taken into consideration?** | **8. Was the data analysis sufficiently rigorous?** | **9. Is there a clear statement of findings?** |
| --- | --- | --- | --- | --- | --- | --- | --- | --- | --- |
| Andersson 2022 | Yes | Yes | Can't tell | Yes | Yes | No | Can't tell | Can't tell | Can't tell |
| Archbold 2002 | Yes | Yes | Yes | Yes | Yes | Can't tell | No | Can't tell | Yes |
| Bartlett 2017 | Yes | Yes | Yes | Yes | Yes | Yes | Can't tell | Yes | Yes |
| Cagulada 2020 | Yes | Yes | Yes | Can't tell | Can't tell | No | Yes | Yes | Yes |
| Doherty 2012 | Yes | Yes | Yes | Can't tell | Yes | No | Can't tell | Yes | Yes |
| Edmondson 2019 | Yes | Yes | Yes | Can't tell | Yes | No | Yes | Can't tell | No |
| Fitzpatrick 2011 | Yes | Yes | Yes | Yes | Yes | No | Yes | Can't tell | Yes |
| Fitzpatrick 2015 | Yes | Yes | Yes | Yes | Yes | No | Yes | Yes | Yes |
| Grandpierre 2018 | Yes | Yes | Yes | Can't tell | Yes | No | Yes | Yes | Yes |
| Hardy 2010 | Yes | Yes | Yes | Can't tell | Can't tell | No | No | Can't tell | No |
| Hilton 2013 | Yes | Yes | Yes | Yes | Yes | Yes | Yes | Yes | Yes |
| Iantaffi 2003 | Yes | Yes | Yes | Can't tell | Yes | Can't tell | No | Can't tell | No |
| Kent 2006 | Yes | Yes | Yes | Can't tell | Can't tell | Yes | Yes | Can't tell | Yes |
| Lindburg 2021 | Yes | Yes | Yes | Yes | Yes | No | Yes | Yes | Yes |
| Lin 2022 | Yes | Yes | Yes | Yes | Yes | No | Yes | Yes | Yes |
| Mather 2011a | Yes | Yes | Yes | Can't tell | Can't tell | No | Yes | Can't tell | No |
| Mather 2011b | Yes | Yes | Yes | Can't tell | Yes | No | Yes | Can't tell | Can't tell |
| Mulla 2013 | Yes | Yes | Yes | Yes | Yes | Yes | Yes | Yes | Yes |
| Preisler 2005 | Yes | Yes | Yes | Can't tell | Can't tell | No | No | Yes | Yes |
| Punch 2011 | Can't tell | Yes | Yes | Yes | Yes | No | Can't tell | Yes | Yes |
| Rich 2013 | No | Yes | Yes | Yes | Yes | No | Yes | Yes | Yes |
| Sach 2005 | Yes | Yes | Can't tell | Yes | Yes | Yes | Yes | Can't tell | Yes |
| Squires 2023 | Yes | Yes | Can't tell | Yes | Yes | Can't tell | Can't tell | Yes | Yes |
| Tanure Alves 2021 | Yes | Yes | Yes | Yes | Yes | Yes | Can't tell | Yes | Yes |
| Terlektsi 2020 | Yes | Yes | Yes | Yes | Yes | No | Yes | Yes | Yes |
| Tierney 2015 | Yes | Yes | Yes | Yes | Yes | No | Yes | Yes | Yes |
| Todorov 2022 | Yes | Yes | Yes | Yes | Yes | No | Yes | Yes | Yes |
| Vieira 2018 | Yes | Yes | Yes | Can't tell | Yes | No | Can't tell | Yes | Yes |
| Watson 2005 | Yes | Yes | Yes | Yes | Yes | Can't tell | No | Can't tell | No |
| Watson 2016 | Yes | Yes | Yes | Yes | Yes | No | Yes | Yes | No |
| Wheeler 2007 | Yes | Yes | Yes | Yes | Yes | No | Yes | Can't tell | Yes |
| Total Yes (n) | 29 | 31 | 28 | 20 | 26 | 6 | 19 | 19 | 23 |
